# Thrombus formation in the noncoronary sinus of Valsalva following primary graft dysfunction

**DOI:** 10.1111/jocs.16482

**Published:** 2022-04-05

**Authors:** John‐Peder Escobar Kvitting, Christian H. Eek, Arne K. Andreassen, Runar Lundblad

**Affiliations:** ^1^ Department of Cardiothoracic Surgery Oslo University Hospital, Rikshospitalet Oslo Norway; ^2^ Institute of Clinical Medicine University of Oslo Oslo Norway; ^3^ Department of Cardiology Oslo University Hospital, Rikshospitalet Oslo Norway

**Keywords:** aorta and great vessels, transplant

## Abstract

We hereby present a case of thrombus formation in the noncoronary sinus of Valsalva following primary graft dysfunction. The case highlights that stagnant and nonpulsatile flow can form thrombi in the noncoronary sinus since this sinus does not have a natural distal runoff.

Primary graft dysfunction following heart transplantation is a calamity. The low cardiac output caused by mono‐ or biventricular dysfunction requires inotropes, pressors, and occasionally temporary mechanical support.^1^ The etiology behind primary graft dysfunction is often multifactorial and not easily predictable pretransplant.^1^, ^2^ The unique anatomy of the sinuses of Valsalva generates a vortical flow pattern in all three sinuses that ensures rapid and efficient closure of the aortic valve as well as facilitating coronary blood flow.^3^ Stagnant and nonpulsatile flow, however, can lead to thrombus formation and potential devastating peripheral embolization.^4^ Thrombus formation in the sinuses of Valsalva is a rare condition, but has been reported in patients with acute coronary syndrome and normal coronary arteriography.^5^ We present a case of thrombus formation in the noncoronary sinus of Valsalva following primary graft dysfunction.

A 66‐year‐old woman with a mutation negative arrythmogenic right ventricular cardiomyopathy and previous multiple implantable cardioverter defibrillators underwent an ABO‐compatible primary heart transplant. The orthotropic transplantation was done through a median sternotomy with standard bicaval and aortic ascendens cannulation. Cold crystalloid cardioplegia was administrated after suturing the left atrium and the pulmonary artery. The transplantation was technically uneventful with a total ischemia time of 92 min (transportation [on‐site procurement] and implantation of 24 and 68 min, respectively). The donor heart was preserved using cold static storage. The donor heart (explanted 60 h after donor brain death, which was due to anoxia) started in sinus rhythm, but did not generate forward flow and intraoperative transesophageal echocardiography showed severe biventricular dysfunction. After prolonged reperfusion peripheral veno‐arterial extracorporeal membrane oxygenation (V‐A ECMO) as well as an intraaortic balloon pump were placed and the patient transferred to the intensive care unit (ICU). The bed‐side echocardiogram in the ICU showed severe biventricular dysfunction. There was no flow in the left main coronary artery, and the aortic valve did not open. On the evening of postoperative day (POD) 1 a coronary arteriography was done that showed sluggish flow in the right and left coronary artery with a thrombus in the noncoronary sinus (Figure 1). On POD 2 the patient was taken back to the operating room and placed on cardiopulmonary bypass, the aortic anastomosis opened and 1.5 × 1 cm thrombus was removed from the noncoronary sinus. A biopsy was taken from the left ventricular septum (that later showed no signs of rejection), the V‐A ECMO circuit was reestablished and the patient transferred back to the ICU. A request for a new organ was made in the Scandiatransplant system. The next 2 days the function of the heart improved dramatically and the patient could be weaned from the V‐A ECMO on POD 4. The etiology behind the recovery of the transplanted heart is uncertain, but after 4 days on V‐A ECMO the biventricular function had greatly improved. Primary graft dysfunction, however, is often resolved within days of the transplant and it is not uncommon that the exact etiology is difficult to identify.^2^ The thrombus in the noncoronary sinus of Valsalva was not the cause of the primary graft dysfunction, but believed to be secondary to low cardiac output leading to stagnant flow in the aortic root. On the latest follow‐up 6 months posttransplant the graft had an ejection fraction of 50% with a small region of hypokinesia in the septum. Right heart catheterization demonstrated a mean pulmonary artery pressure of 28 mmHg, capillary wedge pressure of 19 mmHg and normal cardiac output. The patient was in New York Heart Association Class I.

**Figure 1:**
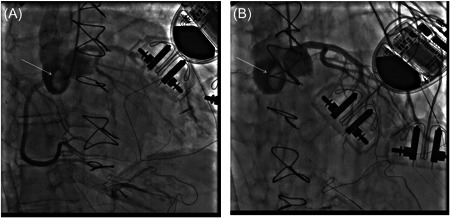
Coronary arteriography of the right (A) and left coronary artery (B). The white arrow marks the thrombus in the noncoronary sinus of Valsalva

Biventricular dysfunction after a heart transplant needing mechanical support can be seen in the setting of primary graft dysfunction.^1^ Thrombus formation in the sinuses of Valsalva is rare, but stagnant and nonpulsatile flow can form thrombi in the noncoronary sinus since this sinus does not have a natural distal runoff.

## AUTHOR CONTRIBUTIONS

All authors have contributed significantly to the manuscript and read and approved the final version of the manuscript.

## CONFLICTS OF INTEREST

The authors declare no conflicts of interest.

## ETHICS STATEMENT

Written informed consent was obtained from the patient for publication of this case report and accompanying images.
